# Fractionating nonword repetition: The contributions of short-term memory and oromotor praxis are different

**DOI:** 10.1371/journal.pone.0178356

**Published:** 2017-07-13

**Authors:** Saloni Krishnan, Katherine J. Alcock, Daniel Carey, Lina Bergström, Annette Karmiloff-Smith, Frederic Dick

**Affiliations:** 1 Centre for Brain and Cognitive Development, Department of Psychological Sciences, Birkbeck, University of London, London, United Kingdom; 2 Department of Experimental Psychology, University of Oxford, Oxford, United Kingdom; 3 Dept. of Psychology, Lancaster University, Lancaster, United Kingdom; Universitat de Barcelona, SPAIN

## Abstract

The ability to reproduce novel words is a sensitive marker of language impairment across a variety of developmental disorders. Nonword repetition tasks are thought to reflect phonological short-term memory skills. Yet, when children hear and then utter a word for the first time, they must transform a novel speech signal into a series of coordinated, precisely timed oral movements. Little is known about how children’s oromotor speed, planning and co-ordination abilities might influence their ability to repeat novel nonwords, beyond the influence of higher-level cognitive and linguistic skills. In the present study, we tested 35 typically developing children between the ages of 5−8 years on measures of nonword repetition, digit span, memory for non-verbal sequences, reading fluency, oromotor praxis, and oral diadochokinesis. We found that oromotor praxis uniquely predicted nonword repetition ability in school-age children, and that the variance it accounted for was additional to that of digit span, memory for non-verbal sequences, articulatory rate (measured by oral diadochokinesis) as well as reading fluency. We conclude that the ability to compute and execute novel sensorimotor transformations affects the production of novel words. These results have important implications for understanding motor/language relations in neurodevelopmental disorders.

## Introduction

The ability to perceive, remember, and then articulate a previously unencountered word is fundamental to our ability to use spoken languages. Indeed, developmental studies have shown that problems with reproducing novel words, or nonword repetition (NWR), can serve as a very reliable marker of language impairment [1–4]. NWR difficulties have also been identified in other neurodevelopmental disorders that have an impact on language development, including Down syndrome [5] and autism [6]. Given this association, there has been much interest in the cognitive and linguistic demands of NWR tasks. Previous research has shown that children’s processing of nonwords is grounded in their existing lexical knowledge, suggesting that there is an influence of long-term memory on nonword repetition [7]. This is sometimes referred to as “phonological proficiency”, or the individual’s knowledge of sub-lexical units in a language. Verbal short term memory is thought to reflect the capacity of the phonological loop of working memory, a system specialised for the temporary maintenance of incoming verbal information [8]. Such capacity is also thought to contribute to individual performance in serial recall tasks like digit span, nonword repetition, and the acquisition of novel vocabulary [9]. Previous research has demonstrated that children's phonological proficiency [10] and verbal short-term memory [1,9] are important for NWR. Yet, at the most basic level, word production is a motor act. The process of reproducing a novel word requires a speaker to identify constituent oral movements that s/he is likely to have produced in other contexts, and use these to plan and execute a novel sequence. When an adult speaker hears and then reproduces a novel word, she draws on decades of experience in selecting, sequencing, integrating, and executing different elements from a honed repertoire of articulatory movements. Perhaps unsurprisingly, when children learn to repeat novel nonwords, they show less consistency in their articulatory coordination across different spoken tokens than do adults learning the same nonwords [11]. Yet, it remains unclear how differences in oromotor proficiency contribute to NWR, above and beyond other ‘higher-level’ cognitive and linguistic skills. Here, we focus on the influence of different aspects of oromotor control on NWR, and explore whether they uniquely contribute to the production of novel words—in addition to the factors such as memory and phonological proficiency that are traditionally thought to play a role in NWR.

### Memory-based accounts of non-word repetition

For historical reasons, the contribution of higher-level cognitive and linguistic factors to NWR is well explored. NWR was originally designed as a test of short-term memory [12–14], with the hypothesis that phonological short-term memory (pSTM)—as indexed by NWR—played a role in language learning. Initial evidence came from neuropsychological cases, which suggested that acquired pSTM deficits hamper novel word learning (reviewed in [9]). In subsequent studies, children with language impairment were found to perform poorly on NWR tasks (reviewed in [1]), and to a lesser extent, on other tasks that tap pSTM, such as digit span. Gathercole et al. [14] also reported a causal link between phonological STM and vocabulary knowledge in typically-developing children. However, other evidence suggests that the link between pSTM and language is not as clear as was initially hypothesised. For instance, Gathercole et al. [15] demonstrated that early pSTM deficits in 5-year-olds did not account for later language deficits in the same children when they were 8 years old. There is also disagreement about the direction of causality between NWR and vocabulary learning [16].

NWR was initially argued to be a ‘pure’ measure of short term memory, in that long-term lexical knowledge could not support the temporary storage of phonological forms [13]. Furthermore, this STM capacity was argued to be causally related to vocabulary acquisition, and used to explain language impairments [12]. A number of studies have now shown that NWR is not a pure measure of STM; rather, as reviewed below, it is clear that many acoustic/phonetic and linguistic skills are associated with NWR. Indeed, NWR may be a sensitive marker for language proficiency, precisely because it taps multiple perceptual, memory, cognitive, linguistic and motor abilities. For instance, performance on NWR tasks is affected by hearing difficulty [17], which leads to difficulty encoding the acoustic form of the word to be reproduced. It is also affected by difficulties in either forming phonological representations or holding these representations in memory [18]. Typically-developing children's NWR is also modulated by their long-term lexical knowledge, as phonological sensitivity [19], and learning to read [20] have been shown to relate to NWR performance. The phonotactic probability of phoneme sequences within the word [7,21] and word-likeness [22] influence NWR. Furthermore, previous studies have used sequential tasks like digit span to assess phonological short-term memory, and a combination of sequential and non-sequential tasks like ‘maze memory’ and ‘visual patterns’ to assess visuospatial short-term memory [23]. More recent studies suggest that *non-verbal* sequencing tasks and phonological memory tasks could impose common demands on procedural learning systems important for learning language [24,25]. Thus, in order to understand how different aspects of memory may influence children's ability to remember and reproduce novel words, it is useful to test measures of both verbal/phonological memory (such as digit span) as well as completely non-verbal memory (for instance, reproduction memory of audiovisual sequences; [26–28].

### Motor contributions to nonword repetition skills

By contrast with the relatively large literature on the relationship of memory and phonology to NWR, the contributions of motor skill to NWR are less well understood. From a clinical standpoint, the co-occurrence of motor and language deficits is particularly important to characterise. Archibald and Gathercole [29] (amongst others) have suggested that there are indeed motor demands beyond pSTM that play a role in NWR. Intriguingly, they found that children with Language Impairment (LI) were less accurate when repeating nonwords like 'fowmoychee' than when recalling the same syllables in the same order, but pronounced in isolation ('fow… moy… chee'), perhaps because of the increase in co-articulatory and speech motor demands (also see [30]). More recently, Reuterskiöld and Grigos [31] have shown that children and adolescents differed on a range of measures such as production accuracy, jaw movement duration, and jaw movement variability, when repeating words and nonwords. This was the case even when the nonwords were carefully matched for number of syllables, stress patterns, linguistic complexity and phonotactic probability, suggesting that long-term motor knowledge of the word was likely to cause this difference. Links between movement rate and language disorders have also been reported; for example, children with dyslexia have been reported to have difficulties producing speeded movements in both manual and oral domains [32–34]. Given that a slower rate of articulation would increase the length of time that words would have to be remembered while being produced, articulatory rates may contribute to estimates of pSTM and NWR skill [35]. However, the method by which speech rate is measured affects its relationship to measures of memory span. For instance, Ferguson, Bowey and Tilley [36] showed that single-word speech rates only accounted for a small proportion of the variance in memory span. In contrast, speech rates derived from multiple-word production loaded onto the same factors as memory span. The authors hypothesised that the more complex words increased memory demands. One means of minimising these demands while estimating articulation rate might be to use oral diadochokinesis (DDK) tasks, which involve articulating the same syllable or series of syllables [32,33,37,38]—a point to which we return later.

Of course, the rate at which an individual child can articulate represents only one facet of potential motoric contributions to NWR skills. Another is the ability to encode a complex sensory signal—like hearing a spoken word or seeing a mouth move—and 'translate' it into a sequence of oral movements—a skill we term '*oromotor praxis*'. A primary demand of NWR is such a recombination and assembly of familiar units of movement into more complex and co-articulated oral sequences.

Links between oromotor praxis and NWR were originally noted in the KE family, where affected members had a point mutation on FOXP2 [39,40]. Affected family members were all more impaired than unaffected members on NWR and oromotor praxis, which was measured by having participants reproduce short sequences of non-speech oral movements like 'open mouth = > round lips = > stick out tongue' [41]. Links between oromotor praxis and NWR have also been observed in children with apraxia of speech; for example, Stark and Blackwell [42] demonstrated that children with language impairment who had difficulties with oromotor praxis also had impairments in NWR. In typical development, we recently found that the ability to imitate novel non-linguistic oromotor sequences predicted the ability to imitate novel, phonotactically legal nonwords in two cohorts of children [43]. This relationship was independent of age, cognitive and language skills, and was robust even when controlling for skill in reading, attentional ability or complex auditory perception.

However, the Krishnan et al. [43] study did not clarify the potential mechanisms underlying this relationship—in particular, whether oromotor praxis was simply acting as a proxy measure for potentially closely-related abilities. For example, there have been suggestions that sign language and lip reading rely on phonological STM [8]. Links between oromotor praxis and language production could also be due to shared demands on more general sequence encoding and reproduction skills [25,44,45]. Equally, they might simply reflect the influence of individual differences in children's rates of articulation, as noted above.

Thus, in the current study, we attempt to fractionate the potential cognitive mechanisms contributing to NWR in children by using a battery of tasks measuring phonological short term memory, non-linguistic sequential memory, reading skill, articulatory rate, and oromotor praxis. We test an independent sample of children in the early school years (ages 5–8), a time of considerable change in language and cognitive skills, with increases in rate of articulation [46,47], memory span [23,48,49], vocabulary size, sentential complexity, and literacy [50]. We analyse the resulting data with particular emphasis on understanding how the mechanisms (putatively) indexed by each task might covary or alternatively uniquely contribute to individual variability in children's NWR skills.

## Methods and procedure

### Participants

Thirty-seven children (22 males) between the ages of 5.4 to 8.6 years participated in the study (see Table 1); none were participants in the Krishnan et al. [43,51] studies. However, this dataset is one of the control groups reported in Krishnan et al. [52]. We excluded all data from one child as the parents reported mild sensorineural hearing loss. All other participants had no history of hearing impairment, language difficulty, or neurological damage (assessed via parental report). All participants orally assented and their parents gave written informed consent. The study received ethical approval from the Birkbeck Research Ethics Committee. Due to a technical fault, we did not record oromotor data from one participant, leaving a total of 35 children (20 males; 5–6 years, *n* = 6; 6–7 years, *n* = 12; 7–8 years, *n* = 9; 8–8.5 years, *n* = 8).

**Table 1:** Task descriptives.

	N	Mean	S.D.	Min	Max	Task Ceiling
***Age***	35	7.1	0.97	5.4	8.6	n/a
***Nonword repetition***	35	12.8	2.5	5.8	16.3	18
***Oromotor Praxis (OM)***	35	93.7	10.1	68	107	120
***- OM gap***	35	45.1	6.2	30	58	60
***- OM no gap***	35	48.6	5.7	33	57	60
***- OM simultaneous***	35	49.9	4.8	41	59	60
***- OM sequential***	35	43.8	6.7	26	54	60
***DDK rate—alternate***	35	0.21	0.02	0.17	0.28	n/a
***DDK rate—sequential***	33	0.72	0.14	0.49	1.01	n/a
***Reading Efficiency***	35	55	14.7	12	80	104
***Digit Span Trials Correct***	35	28.7	4.8	21	41	54
***Total AV sequence score***	35	45.3	13.6	14	68	210

### Experimental tasks

#### 1) Nonword repetition (NWR)

The NWR subtest was taken from the Comprehensive Test of Phonological Processing (CTOPP; [53]). The 18 nonwords (ranging from one to six syllables) were recorded by a native British-English speaker and presented over headphones (see also [20,54]). Children were asked to repeat the word just heard. Three practice trials were followed by the 18 test items. The CTOPP’s nonwords obey English phonotactics, and contain no consonant clusters while varying considerably in their phonological complexity. We did not use simple ratings of correct/incorrect as we found such ratings to be inadequate at capturing variance in previous samples [43]. Instead, fractional scores were awarded on the basis of accuracy using a categorical coding scheme. Scores for each word ranges between 0–1 in increments of 0.25. For an entirely accurate repetition, 1 was awarded, and for an entirely incorrect production, 0 was awarded. In the CTOPP, there are nine words with less than four syllables, and nine words with four or more syllables. For words with 2–3 syllables, we deducted 0.5 if a syllable was incorrectly articulated. For words composed of 4–6 syllables, 0.25 was deducted for every syllable incorrectly articulated or articulated out of order. Two exceptions were made within our coding scheme. In both bisyllabic words of the CTOPP, common errors were observed at the phoneme level (the substitution of the final phoneme, ‘naigone’ instead of ‘naigong’, and the distortion of the vowel ‘oo’ in ‘woodipe’). If a child made an error of this nature on these two words alone, 0.75 was awarded to capture the subtlety of these errors. This coding scheme is identical to the one used in previous paper [43]. After initial scoring, a different researcher independently scored the audio recordings of the children’s responses using the same scale. Inter-rater reliability was >0.8 over all nonwords.

#### 2) Oromotor praxis

**Fig 1:**
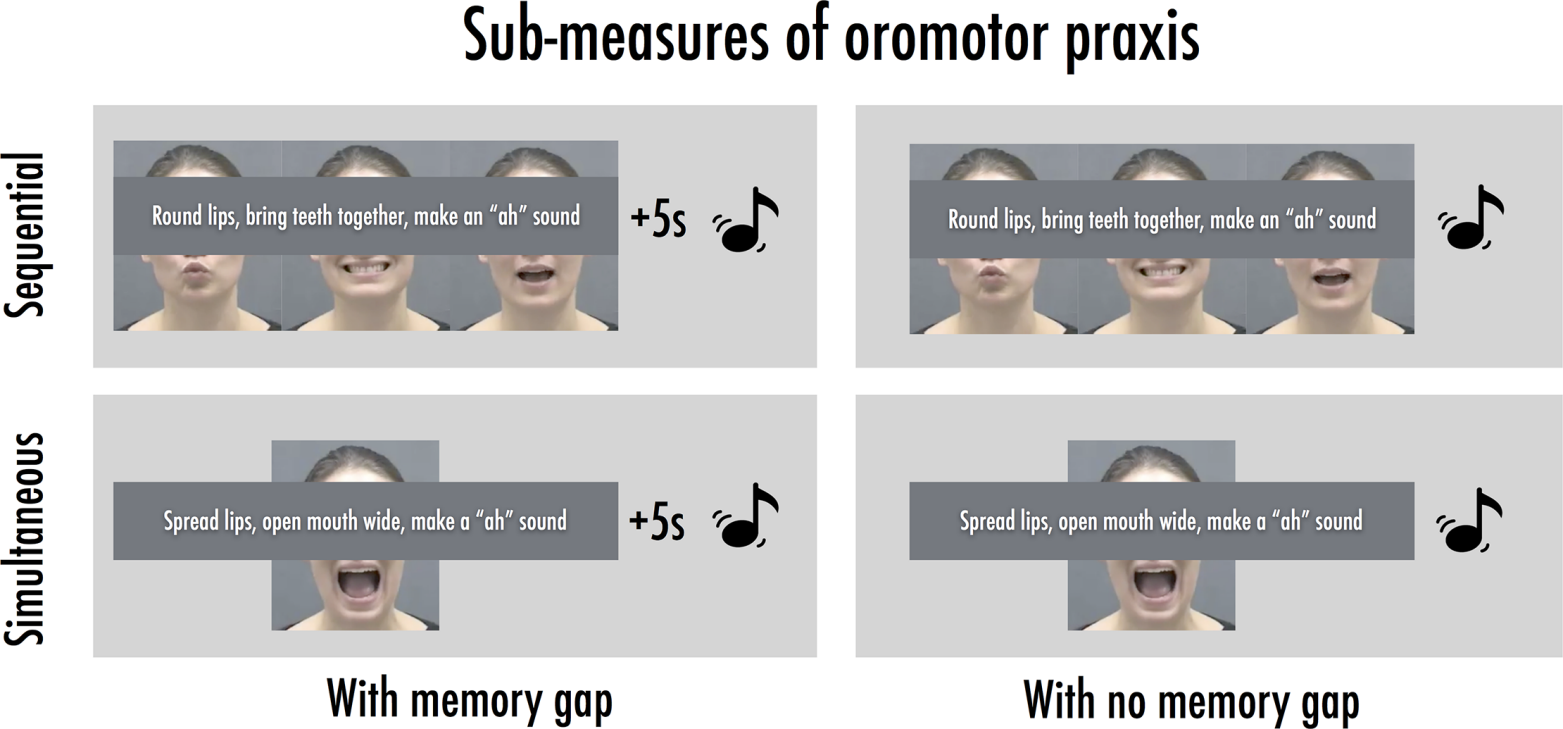
Schematic of the sub-measures of the oromotor praxis task.

Children were video-recorded as they imitated video stimuli of a researcher making non-linguistic oral movements occurring in a non-linguistic context (adapted from [39]). Each trial involved a set of oral movements produced using three articulators (for example, “opening mouth”, “rounding lips”, “sticking out tongue”). Some of these movements did involve listening to a sound, for example, the researcher said ‘a’ as she stuck out her tongue. However, a strategy that involved encoding the sound alone was insufficient to encode and retrieve the movements sequences because the same sound could be associated with different movements (for instance, “a” could correspond to “opening mouth” or “sticking out tongue”). Children had to imitate the movements perfectly, producing a sound if there was one present. There were 20 test trials. In half of the trials, the oral movements were presented simultaneously, and in the other half, sequentially. In the case of a simultaneous movement, all three movements were completed at the same time, necessitating action from all three articulators at one point of time. For sequential movements, however, participants had to imitate the movements one after the other, in the sequence they were presented. The sequential and simultaneous conditions were chosen on the basis of research on acquired speech and language disorders (see [39]). Each condition was preceded by three practice trials when children received verbal feedback on their performance. The sequential and simultaneous conditions were crossed by the presence of a memory gap in half the trials (Fig 1). This gap condition was introduced on the basis of previous research showing that such gaps can reveal subtle individual differences not evident when using the standard task [55]). Children were asked to respond only after they heard a xylophone sound—in the trials with a memory gap, this sound was played 5 seconds after stimulus presentation. In the other trials (‘no gap’), the sound was played immediately when the video stimuli ended. Participants imitated five movements from each of these four conditions and the order of these conditions was counterbalanced across participants.

Each oral movement was scored on a scale of 0–2 (0 representing an inaccurate imitation or no movement, 1 awarded for a partially accurate imitation or when a movement was made in the wrong order, and 2 for a perfect imitation). Scores could thus range between 0–6 per trial, and between 0–120 for the entire task. Thirty percent of the videos were recoded by an independent rater, with inter-rater reliability > 0.8 over participants and conditions (simultaneous, sequential, with/without memory gap).

#### 3) Digit span

The digit span test from the Working Memory Test Battery for Children (WMTB-C; [56]) was administered to assess phonological STM. This test has been found to correlate with NWR [57]. The child is asked to remember a sequence of digits in their order of presentation. At each level, the number of digits in a sequence increases. Each level has six sequences, with the child continuing to progress through the levels until four sequences are answered incorrectly. We used the total number of trials correct as the child’s score; as the digit span measure includes 6 sequences per level with a maximum level of 9, the maximum number of correct trials is 54.

#### 4) Audiovisual sequence reproduction

Non-linguistic auditory-motor novel sequence reproduction was tested using an audiovisual task developed in-house and presented on an iPad. The task is similar to a popular, interactive children’s game and is thought to tap visuospatial working memory (‘SIMON’–[26]). We used this task to identify individual differences in the reproduction of novel audiovisual sequences. While we refer to this task as relying on sequential working memory, it is important to note that the task makes demands on multiple cognitive skills, and therefore performance will reflect contributions from domain-general sequencing ability, working memory, reproduction ability/imitation skill, and visuospatial STM.

The gaming interface simulated four buttons arranged in a circle. Four tones (262 Hz [C4]; 327.5 Hz [E4]; 393 Hz [G4]; 524 Hz [C5]) were used, each being uniquely paired with a single button. Tone/button pairings were randomly chosen, but fixed over the entire game. During play, each button was illuminated and the associated tone played simultaneously. While the auditory information associated with each button was distinctive in this task, in previous testing with adults, we have observed that using the same tone for all buttons (to control for attention) did not lead to differences in performance. The sequences were constructed such that every tone had an equal probability of occurrence regardless of its predecessor (e.g., no first-order temporal correlations).

Children had to reproduce a sequence of tones presented by the interface, with each tone played along with an associated button that lit up when the tone sounded. On a given trial, the interface presented a sequence, and then cued the participant to respond by showing an unobtrusive icon at the centre of the display. The first trial was always a single tone. Participants imitated sequences in their entirety by pressing the buttons in the order they appeared. If the trial was imitated correctly, the length of the sequence incremented by one item (button + tone) and a new trial commenced. The participant’s score reflected the number of items correctly sequenced. The goal of the game was to achieve as high a score as possible. The score for each trial was displayed on the bottom of the screen and updated after each trial. A sequence was complete either when the participant reached a length of 21 items, or when s/he reproduced the sequence incompletely, out-of-order, or incorrectly. After completing a sequence, participants took a short break before beginning the next one.

At the beginning of the experiment, participants completed three practice sequences of six items. If a child reproduced less than four items on either practice, the three practice sequences were re-run until a minimum of four items was achieved for at least two practice sequences (the practice criterion also allowed us to ascertain that visual acuity and manual motor demands did not affect performance). The ten test sequences were then presented. The maximum length of the sequence (akin to level in the digit span task) was set at 21 (a level which only a few adults reach). The child’s score was the total sum of the scores they attained across the ten sequences; with 10 sequences total, the maximum possible score is 210.

#### 5) Reading

We administered the sight word reading efficiency subtest for familiar words from the Test of Word Reading Efficiency (TOWRE:[58]). This is a simple fluency test, involving reading a list of progressively more complex words within 45 seconds. The child’s score is simply the number of words accurately read. TOWRE scores have previously been shown to be a good predictor of NWR [20].

#### 6) Alternating and sequential diadochokinetic (DDK) speech rate

The task gives an estimate of the child's rate of articulation. The child was asked to repeat the syllables [pa], [ta] and [ka] and the trisyllablic sequence [pataka], 12 times as fast as possible and on a single expiration [59]. The task was first modelled by the experimenter, followed by a practice trial. There were test trials for each of the four conditions ([pa], [ta], [ka], [pataka]). Responses were recorded in a sound-attenuated room using a digital recorder. After manual segmentation, the duration of each trial was automatically extracted using Praat. As some children did not complete 12 uninterrupted repetitions, we analysed trials with a minimum of 11 accurate repetitions. The duration of the shortest accurate performance from these three trials was retained for further analysis.

The average length of each syllable or syllable sequence was calculated by dividing the total duration of articulation by the number of syllables (or syllable sequence in the case of [pataka]) in each production. The alternating DDK rate was calculated by averaging the rates for each of the single syllables [pa], [ta] and [ka]. The sequential DDK rate was that for the [pataka] sequence.

### Plan for analysis

We will first assess simple pairwise correlations between NWR and demographic variables (age/ gender). As we use raw scores in all these analyses, age will be retained as a predictor if it is correlated with NWR.

We will then run simple pairwise correlations with all our predictors (oromotor praxis, reading efficiency, digit span, audiovisual sequencing, and the two DDK measures). We expect that these measures will be correlated to each other. Consequently, to assess if a predictor makes a unique contribution to NWR, we will present results from a series of regression models to understand how the abilities underlying each task might uniquely contribute to NWR skills. In order to constrain the possible search space of regressors, only predictors (including age) that are pairwise correlated with NWR at r > = 0.3 (p < 0.1) will be included. To avoid overfitting given the sample size, all models will be constrained to have no more than three predictors. No correction for multiple comparisons will be applied to this set of models, as the rationale for the series of models is to explore which predictor subset contributed the most variance, rather than making inferences on each model. Regression models will be bootstrapped (10,000 replications) to control for the influence of any potential outliers and non-normal distributions. The bootstrap is a data-based simulation method for statistical inference [60]. Confidence intervals for a statistic of interest may be incorrectly estimated for a small sample. Bootstrapping allows one to build a population by repeatedly resampling data from the same sample. This allows for the empirical estimation of the confidence intervals and standard errors associated with a parameter of interest [61]. To assess how each predictor contributes to the model, we will conduct permutation analyses on the bootstrapped regression models. Here, the dataset is held constant but the values are randomly permuted relative to the core variable. As all permutations are equally likely under the null hypothesis of no association, the null distribution underlying p-values can be empirically estimated. This set of models will allow us to assess which variables make consistent and separable contributions to NWR scores. We will also confirm the results of this approach by conducting a stepwise regression (where the choice of predictors is data driven).

Finally, if oromotor praxis is a unique predictor of variance in NWR scores, we will explore if all sub-scales of oromotor praxis relate to NWR. We will compare the slopes of these regression lines to assess if any sub-scale makes a distinct contribution.

## Results

### Age and gender effects on NWR

These analyses were conducted to assess the effects of these demographic variables on NWR. Age was significantly associated with NWR scores (*r* = 0.4039, *p* = 0.0161); girls' NWR scores (*M* = 13.7, *SD* = 1.8) were on average higher than boys' (*M* = 12.06, *SD* = 2.7; *t*(32.4) = 2.15, *p* = 0.0388). The interaction between age and gender was non-significant (p > 0.2). Given the correlation between age and NWR, and our use of raw scores in all the analyses below, we also retain age as a predictor when assessing the influence of task on NWR.

### Pairwise correlations between NWR and other tasks

**Fig 2:**
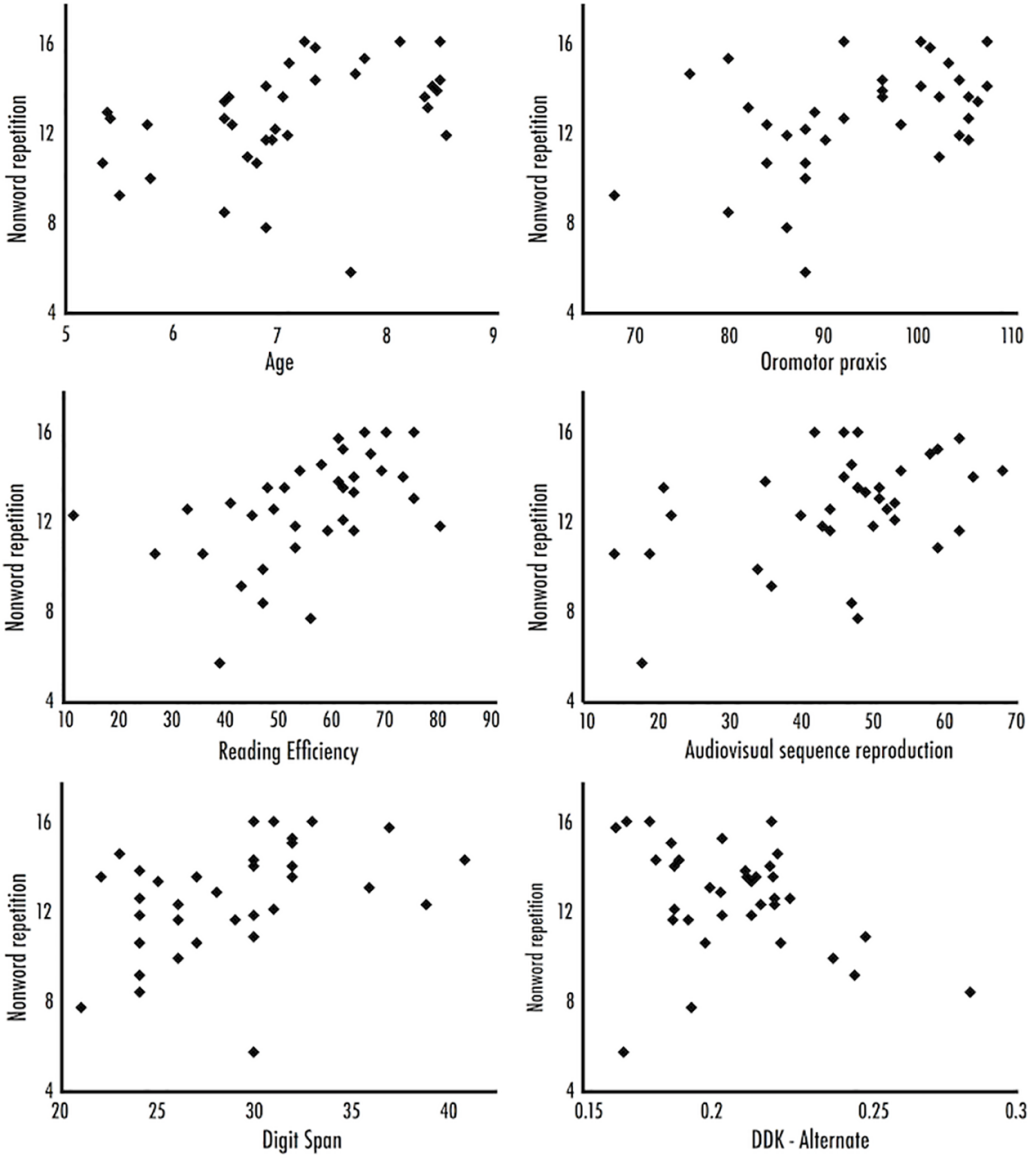
Scatterplots depict the relationship between raw nonword repetition scores and each of the predictors. Predictors include age; reading efficiency; digit span trials correct; oromotor praxis; total audiovisual sequence reproduction score and alternating DDK rate.

As an initial step, we calculated pairwise correlations between scores for NWR and all other tasks (see **Table** 1 for descriptive statistics on each task). As shown in **Table** 2 and **Fig 2**, NWR was pairwise positively correlated with all tasks at *p* < 0.05 (oromotor praxis, *r* = 0.43; reading efficiency, *r* = 0.48; digit span, *r* = 0.41; audiovisual sequencing, *r* = 0.44) but significant correlations were not observed for the two diadochokinesis (DDK) measures. The DDK-alternate task marginally correlated *(r* = -0.3302, *p* = 0.0527, where faster rates of articulation were associated with higher NWR scores), and DDK-sequential was not significantly correlated. There were three outliers in the DDK-alternate sub-scale; when excluded, the correlation between DDK-alternate and nonword repetition was significant. However, we did not have any *a priori* reason to exclude these children, so they were retained for further analyses. We return to this issue in the discussion. Unsurprisingly, there was also considerable shared variance over all tasks; when all variables (including NWR) were included in an exploratory principal components analysis, weighting on the first principal component was roughly equal over all tasks, and accounted for 47% of all variance.

**Table 2:** Pairwise correlations over tasks.

	Age	NWR	OM	DDK -alt	DDK- seq	Reading Efficiency	Digit Span	Max AV seq
***Age***	1							
***NWR***	.40*	1						
***OM***	0.23	.43**	1					
***DDK -alternate***	-.41*	-.33	-.35*	1				
***DDK—sequential***	-.22	-.23	-.30	.50**	1			
***Reading Efficiency***	.75**	.48**	.30	-.32	-.23	1		
***Digit Span*** ***Trials Correct***	.37*	.41*	.27	-.48**	-.18	.34*	1	
***Total AV sequence score***	.21	.44**	.25	-.21	-.09	.54**	.39*	1

** p < 0.01

* p < 0.05

#### Unique contributions of different skills to NWR

The main thrust of the study was to understand how oromotor praxis, reading proficiency, phonological and audiovisual short-term memory, and overall speech rate might account for inter-individual variation in NWR skills. As detailed above, we tested these contributions by creating a set of regression models based on combinations of task regressors along with chronological age (for details on this approach, see [43]. We included tasks that were pairwise correlated with NWR at r > = 0.3 (p < 0.1); this eliminated only DDK-sequential as a regressor. This approach yielded a set of 41 models (6 models included only one predictor, 15 models included all unique combinations of two predictors, and 20 models that included all unique combinations of any three predictors, for instance, oromotor praxis, reading efficiency, DDK-alternate). Models are presented ranked in descending order in terms of total NWR variance accounted for (see **Table** 3**)**. The models clearly showed that several underlying abilities were uniquely associated with inter-individual variability in NWR, albeit to different degrees. *Oromotor praxis* was included in every one of the 10 top-ranked models (total *R*^*2*^_*adj*_ from 0.26 to 0.31), and accounted for significant unique variance in 5 of 10 models. Importantly, this set of top-ranked models included single models where every other regressor was included—e.g., the unique NWR variance predicted by oromotor praxis was not accounted for by any other task.

**Table 3:** Models (one per row) ranked by R^2,^ including all combinations of the six tasks with a maximum of three predictors in any model. Each task column shows the permutation-based estimated probability that the task contributes unique variance to the regression model.

Rank	*Whole model R*^*2*^_*adj*_	Whole-model *p*	Oromotor praxis	DDK Alternate	Digit Span Trials Correct	Audio-visual sequence score	Reading fluency	Age
**1**	***0*.*313***	**0.000**	**0.047**			**0.042**		*0*.*076*
**2**	***0*.*309***	**0.001**	*0*.*070*		0.148		**0.033**	
**3**	***0*.*297***	**0.005**	**0.050**			0.156	*0*.*061*	
**4**	***0*.*285***	**0.002**	**0.038**				**0.012**	
**5**	***0*.*282***	**0.001**	*0*.*051*		0.155	*0*.*075*		
**6**	***0*.*273***	**0.001**	*0*.*065*	0.461			**0.020**	
**7**	***0*.*267***	**< .0001**	**0.041**		0.130			0.114
**8**	***0*.*265***	**0.000**	**0.045**				**0.039**	0.554
**9**	***0*.*263***	**0.001**	**0.025**			**0.023**		
**10**	***0*.*261***	**0.000**	*0*.*062*	0.314		**0.036**		
**11**	***0*.*260***	**0.003**			0.135	0.235	*0*.*051*	
**12**	***0*.*259***	**0.003**			*0*.*072*		**0.016**	
**13**	***0*.*257***	**0.000**			0.216	**0.049**		0.090
**14**	***0*.*251***	**0.000**				**0.020**		**0.040**
**15**	***0*.*245***	**0.000**		0.351		**0.031**		0.085
**16**	***0*.*244***	**0.008**		0.244		0.122	*0*.*061*	
**17**	***0*.*243***	**0.005**		0.532	0.131		**0.025**	
**18**	***0*.*242***	**< .0001**	**0.022**					**0.044**
**19**	***0*.*236***	**0.002**	**0.028**		**0.047**			
**20**	***0*.*236***	**0.005**			0.088		**0.016**	0.891
**21**	***0*.*235***	**0.016**				0.101	**0.023**	
**22**	***0*.*233***	**0.001**				**0.049**	0.350	0.143
**23**	***0*.*225***	**< .0001**	**0.036**	0.549				*0*.*075*
**24**	***0*.*222***	**0.005**		0.211			**0.012**	
**25**	***0*.*216***	**0.006**	**0.043**	0.614	*0*.*075*			
**26**	***0*.*215***	**0.002**			*0*.*077*	**0.038**		
**27**	***0*.*212***	**0.001**		0.303	0.200	**0.042**		
**28**	***0*.*211***	**0.009**					**X**	
**29**	***0*.*206***	**0.002**		0.121		**0.019**		
**30**	***0*.*198***	**0.011**		0.238			**0.014**	0.892
**31**	***0*.*194***	**0.002**			*0*.*063*			*0*.*067*
**32**	***0*.*191***	**0.009**					**0.010**	0.551
**33**	***0*.*176***	**0.007**	**0.025**	0.211				
**34**	***0*.*175***	**0.004**		0.552	0.104			0.106
**35**	***0*.*170***	**0.007**				**X**		
**36**	***0*.*164***	**0.007**	**X**					
**37**	***0*.*145***	**0.004**		0.221				*0*.*052*
**38**	***0*.*143***	**0.007**			**X**			
**39**	***0*.*141***	**0.017**		0.285	**0.049**			
**40**	***0*.*138***	**0.001**						**X**
**41**	***0*.*082***	0.113		X				

Bolded p-values are p < 0.05; italicised p < 0.08. The X’s denote models where there is only one predictor (the one indicated by X), and therefore a predictor cannot be uniquely significant.

*Reading fluency* (as measured by TOWRE) and *audio-visual sequence reproduction* skill both appeared in half of the 10 top models, with reading fluency accounting for significant unique variance in 4 of these, and audio-visual sequence reproduction in 2 of 10. All three tasks also contributed unique variance to several models apiece in the 11th-20th ranked models. With regard to reading fluency, it is important to note that scores were strongly correlated with age (*r* = 0.75), so some of the effects of reading fluency may well be due to age-related improvements in NWR. *Chronological age* itself accounted for significant unique variance in two of the top-ranked 20 models, and in particular was a marginally significant factor in the very top-ranked model.

Somewhat unexpectedly, *digit span* only contributed unique variance to one model in the top-ranked 20—namely the model including oromotor praxis (which also contributed unique variance). Finally, *speech rate* (alternating diadachokinetic speed) did not account for unique variance in any of the well-performing models.

As a confirmatory analysis, we conducted a stepwise regression using penalised-likelihood criteria to pick the best set of regressors. The model selected using minimum Akaike’s Information Criterion (corrected) included only oromotor praxis and reading efficiency (*R*^*2*^_*adj*_ = 0.2846, *p* = .0019), with both contributing unique significant variance in the model (*p* < 0.05).

#### Sub-measures of oromotor praxis (see Table 4)

Given that oromotor praxis was the most robust predictor of NWR variance, we asked how finely we could fractionate the contribution of oromotor praxis to NWR variance by running pairwise correlations between oromotor subscores and NWR. There were significant correlations between NWR and simultaneous oral movements (*r* = 0.4637, *p* = 0.0050) and movements after a memory gap (*r* = 0.4037, *p* = 0.0162), with marginal correlations for sequential movements (*r* = 0.3196, *p* = 0.0613) and movements made in the absence of a memory gap (*r* = 0.3241, *p* = 0.0575). To compare if the slopes of these regression differed, we ran 4 regression models (NWR with each sub-score, OM with gap, without gap, simultaneous, and sequence), and compared the betas of each using the procedure outlined in Paternoster et al. [62]. Despite the variability in r-values, there was no significant difference (*z* < 1.2) between the regression coefficients for each sub-measure and NWR. Finally, to check whether the 'memory gap' in the oromotor praxis task was acting as an implicit measure of working memory, we first calculated the difference in accuracy between the gap and no-gap scores for each participant. This allowed us to assess the extent to which an individual’s performance worsened when the gap was introduced. We then tested whether the gap minus no-gap difference was correlated with digit span or audiovisual reproduction skill: neither correlation was significant (p > 0.7).

**Table 4:** Pairwise correlations across sub-scales of oromotor praxis and experimental measures.

	OM Gap	OM No gap	OM Simultaneous	OM Sequential
***OM Gap***	1			
***OM No Gap***	.42*	1		
***OM Simultaneous***	.77**	.61**	1	
***OM Sequential***	.73**	.80**	.51**	1
***Age***	.17	.22	.34*	.10
***NWR***	.40*	0.32^+^	.46**	.32
***DDK -alternate***	-.30	-.30	-.55**	-.13
***DDK—sequential***	-.28	-.23	-.28	-.25
***Reading Efficiency***	.24	.26	.40*	.17
***Digit Span*** ***Trials Correct***	.19	.27	.46**	.08
***Total AV sequence score***	.22	.21	.28	.17

** p < 0.01

* p < 0.05

## Discussion

In a previous paper, we established that oromotor praxis was related to nonword repetition (NWR), beyond several linguistic and non-linguistic skills [43]. However, that initial experiment was not designed to disambiguate the potential psychological mechanisms underlying the contribution of oromotor praxis to NWR ability. Above all, it did not establish whether oromotor praxis was simply a proxy measure for other hypothesised contributors to NWR, particularly short-term memory or sequencing ability. It is the disambiguation of mechanisms underlying NWR that the present paper addresses. In this group of younger school-age children, we find NWR skills were associated most reliably with oromotor praxis, reading fluency, and audiovisual sequence reproduction accuracy. The association between oromotor praxis and NWR abilities was not simply due to shared variance with either phonological STM, working memory or age, thus suggesting that it has a unique role in the process of remembering and reproducing novel words—a skill crucial for language development.

This finding has implications for understanding the processes that underlie developmental language disorder and delay. We speculate that difficulties in oromotor praxis may be particularly apparent at the earliest stages of learning to produce a word, but these difficulties may be less important once the same word is practised and familiar. This would suggest that NWR and oromotor praxis could be underpinned by a common need for utterance planning [63], or creating action goals for the purpose of speaking, in a novel context. While it is unclear if initial difficulties in oromotor praxis could act as a constraint on other language skills, reproduction or imitation of words is a strategy used by adults to learn [64]. This is likely due to the opportunity to develop a richer sensorimotor representation of the word. Consequently, relative weakness in oromotor praxis—probably in tandem with relative weakness in other abilities such as sequential memory encoding—may limit the learning strategies available to children and therefore predispose them to have language difficulties. This would fit with findings of language impaired children's particular sensitivity to changes in motoric demands during word production: for instance, Archibald, Joanisse, and Munson [65] recently showed that when typically developing children and children with LI articulated complex nonwords with and without motoric constraints (induced by a bite block), only the children with LI showed more impairment in the constrained task. This suggests that articulatory planning may be a 'weak link' in these children. However, it will be important to disambiguate which aspects of oromotor ability and articulation might be affected across different populations, since motor speed, sequencing and imitation can be differently affected in reading and language disorders [34].

Given that NWR requires the co-ordination of familiar units of movement into more complex oral utterances, it is perhaps unsurprising that individual differences in NWR were reliably associated with our non-linguistic probe of this process. Nonetheless, it is noteworthy that this association is not due solely to underlying shared differences in children's memory skills, as has previously been hypothesised. Rather, individual differences in sequential memory encoding and reproduction skill (as measured by the SIMON task) appear to make additional and unique contributions to children's NWR abilities. Interestingly, this non-linguistic sequential memory task turned out to be a more reliable predictor of NWR skills than digit span, which is classically used to assess auditory verbal short-term memory [66,67].

What aspects of the sequential memory task are important for this relationship? In particular, is the task measuring a form of auditory non-verbal working memory? To get at this question, we have since tested a version of the task where auditory information was non-informative. While the version used in the present study assigned a unique tone to each button, in the alternative variant, only one tone frequency was used for all buttons. Contrary to our expectations, we observed no difference in the average sequence length obtained by adults in the one-tone or four-tone condition, suggesting that the task primarily measures visual-motor rather than auditory-motor memory encoding skill [68]. Furthermore, other studies that have used a similar version of this task report that performance is moderately to strongly correlated with working memory measures such as backwards digit recall and visuospatial performance [26,27]. This suggests in turn that the unique contribution of memory in NWR might not be specific to the auditory or phonological domains. Rather, the capacity to attend to and encode *sequential* information in the auditory or visuospatial domain might be the factor making a unique contribution to NWR. Sequencing ability in both auditory and visuospatial domains have also been linked to language impairment [25,29,69,70]. These results suggest that sequential memory is important for aspects of language even in typically developing children.

Returning to the more general question of the role of motor and articulatory skill in NWR, our results appeared to suggest that oromotor praxis made a contribution to NWR, while DDK did not. This was particularly striking as the DDK tasks involve articulation of speech sounds while the oromotor praxis task does not involve speech sounds or practised movements. Furthermore, the nature of presentation was auditory in the case of DDK, and audiovisual in the case of oromotor praxis. However, as noted previously, the seeming distinction between NWR-oromotor praxis and NWR-speech rate relationships (especially as assayed by DDK-alternate) should be taken as preliminary. First, oromotor praxis and DDK-alternate were significantly correlated, with those scoring higher on praxis also responding faster on DDK tasks. Second, as we noted in the results, there were 3 outliers in the pairwise correlation between DDK and NWR; when these datapoints are removed post hoc, a significant relationship between the two measures does emerge. Thus, we should tread cautiously in interpreting this finding. This is particularly true given that both tasks require the transformation and oral execution of sensorimotor movements. Indeed, the cyclic movement patterns in DDK may be the building blocks for performing complex oromotor praxis (or vice versa). Future studies with clinical populations where we might expect to see dissociations between oromotor praxis and oromotor speed (for example, autism, [71–73], may help address how different aspects of oromotor skill relate to language.

Finally, we found that reading efficiency was associated with NWR. A causal influence of reading on NWR has been previously claimed [20] and our findings could certainly reflect this influence. However, in our dataset, there may be an additional reason for the relationship between NWR and reading efficiency. As we did not control for verbal age or vocabulary ability, the reading measure is likely to reflect the contribution of not only reading but also acting as a proxy for these verbal abilities. For example, it is known that this reading efficiency measures correlates with vocabulary knowledge. Consequently, our speculation is that reading efficiency captures the lexical/phonological dimension of NWR. This fits well with the lexical restructuring hypothesis proposed by Metsala and Walley [74], which suggests that as children’s vocabulary increases, there is pressure on the system to re-organise and differentiate words that are close together in phonological space. This causes finer segmental structure to emerge. Vocabulary-driven changes of phonology have also empirically demonstrated by de Cara and Goswami [75], who show that 5-year-olds with higher vocabulary ages perform better on rime oddity tasks (an index of phonological awareness) relative to those with lower vocabulary ages. Learning to read, in addition to improving vocabulary, could also lead to children encountering many more spelling-sound mappings, which would also exert pressure on phonological structure to re-organise. In other words, we think that reading efficiency on this task captures an element of verbal or language ability.

We have argued that NWR captures important aspects of oromotor ability, language ability and sequential memory that are crucial for language production, and these are particularly relevant to consider if NWR is used in screening for LI. We note that the directionality of these relationships remains uncertain, and that further research with younger children is also needed to assess the developmental trajectory of oromotor abilities and how they influence—and are influenced by—language growth over time. Indeed, the relationship between NWR and oromotor ability is likely to be bidirectional in the school years. As an example, expanding vocabulary might exert increased pressure on the motor system that then responds to these demands [76]. By studying the trajectory of co-developing motor, sequential, and language skills, we should be able to reveal the common developmental pathways that underlie differences in linguistic ability.
